# Evaluation of *ebony* as a potential selectable marker for genetic sexing in *Aedes aegypti*

**DOI:** 10.1186/s13071-025-06709-y

**Published:** 2025-02-25

**Authors:** Katerina Nikolouli, Austin Compton, Zhijian Jake Tu, Kostas Bourtzis

**Affiliations:** 1 Insect Pest Control Laboratory, Department of Nuclear Sciences and Applications, Joint FAO/IAEA Centre of Nuclear Techniques in Food and Agriculture, IAEA Laboratories, 2444 Seibersdorf, Austria; 2 Department of Biochemistry, Virginia Tech, Blacksburg, VA24061 USA; 3 Fralin Life Sciences Institute, Virginia Tech, Blacksburg, VA24061 USA

**Keywords:** *Ebony*, Genetic sexing strain, *Aedes aegypti*, Sterile insect technique

## Abstract

**Background:**

*Aedes aegypti* is expected to invade previously unoccupied areas, mainly due to the climate change, the increase in travel and trade activities and the continuous transformation of the rural environment into urban areas. The sterile insect technique (SIT), which relies on the mass production and release of sterile males, is an environmentally friendly approach that can be applied for population control of *Ae. aegypti.* SIT programs can be greatly benefited by a genetic sexing strain (GSS) and a reliable sex sorting system to minimize any accidental female release. Visually detectable or conditionally lethal selectable markers can be used for the development of new GSSs. In this study, we evaluated the suitability and competence of a mutant *Ae. aegypti ebony* strain for the development of a new GSS. The *ebony* gene is known to be involved in the pigmentation pathway of several dipteran insects, including *Ae. aegypti*.

**Methods:**

An *ebony* gene knockout was developed though CRISPR/Cas9 mutagenesis. G_0_ individuals with the desired phenotype were crossed, and progeny were screened in every generation. PCR and sequencing were performed using gDNA from a pulled leg to determine the mutant genotype. Quality control tests, including pupae and adult recovery rates, male sex ratio and fecundity, were applied to the *ebony* mutant line to determine whether the mutation confers any fitness cost.

**Results:**

An *Ae. aegypti ebony* knockout mutant carrying a 5-bp deletion was obtained, which presented darker head and siphon phenotypes at the larval stage. However, genetic analysis revealed that this *ebony* mutation results in incomplete penetrance and variable expressivity. The establishment of a pure *ebony* mutant line was not possible because of the fitness costs conferred by the mutation.

**Conclusions:**

In this study, the adequacy and suitability of the *ebony* gene as a selectable marker for the development of a GSS in *Ae. aegypti* were assessed. Despite its clear phenotype early in larval development, the homozygous mutant line presented phenotypic inconsistency and loss of fertility. These drawbacks clearly indicate that this particular mutation is not suitable for the development of a new GSS. Nonetheless, it cannot be excluded that a different mutation will lead to a different expression and penetrance profile and a viable homozygous mutant line.

**Graphical Abstract:**

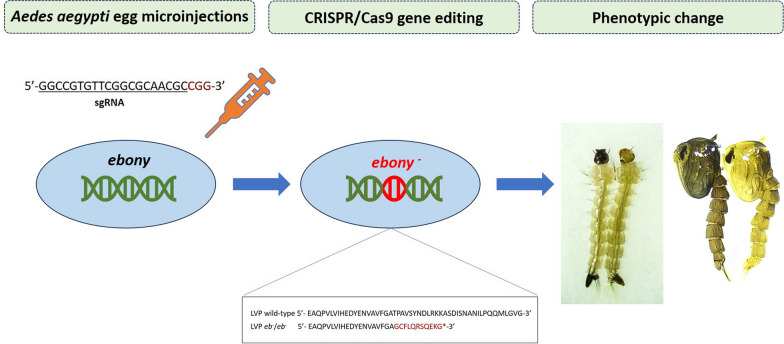

**Supplementary Information:**

The online version contains supplementary material available at 10.1186/s13071-025-06709-y.

## Background

*Aedes aegypti* L. (Diptera: Culicidae) mosquitoes are responsible for the transmission of several vector-borne diseases attributed to arboviruses, including Zika, dengue, yellow fever and chikungunya.[1–4] Mosquito population control methods currently rely on insecticide application, an approach that has led to the development of resistance, which has severely jeopardized efforts to control the vector population [5]. Therefore, there is a growing interest in environmentally friendly approaches, such as the sterile insect technique (SIT), which relies on the mass production and release of sterile males [6, 7]. SIT has already been applied in the field to control mosquito populations, with remarkable results [8–10]. However, sex separation and female elimination remain major obstacles in the large-scale application of SIT against mosquitoes [11].

Accidental release of female mosquitoes can increase not only biting nuisance but, most importantly, the spread of arboviruses since females bite and transmit diseases. Regarding *Wolbachia*-infected mosquitoes, which are used in the frame of the incompatible insect technique (IIT), any accidental release of *Wolbachia*-infected females can jeopardize the success of the program since a population replacement is possible [12]. To mitigate this risk, the combination of IIT with SIT has been proposed as it ensures that any inadvertently released *Wolbachia*-infected females are irradiation-sterilized [13]. Regardless of the approach (SIT or SIT/IIT), scientists are hunting for reliable sex sorting systems that will allow for efficient and error-free male-female separation at the earliest developmental stage possible [14, 15].

Sex sorting can be performed either based on inherent species characteristics or by developing genetic sexing strains (GSSs). Development of a GSS using classical or neo-classical genetic approaches requires a visually detectable or conditionally lethal selectable marker that is detected either by population screening or induction of mutagenesis [16, 17]. The wild-type allele of this marker is linked to the Y chromosome or to the sex-determining genetic locus of the species [16]. *Aedes aegypti* males are defined by a dominant male-determining locus (M locus) on chromosome 1. While males are heterogametic (Mm), females are homogametic (mm) and lack the M-locus [18, 19]. Selectable markers that reside on chromosome 1 are strong candidates for the development of a GSS in which males are heterozygotes and express the “wild-type” phenotype, while females are homozygotes for the recessive alleles and express the mutant phenotype [16]. Two *Ae. aegypti* GSSs based on genes related to eye color are currently available [20]. Both genes (*red-eye* (*re*) and *white-eye* (*w*) markers) are located on chromosome 1 and are linked to the M-locus, showing complete penetrance and expressivity [21, 22].

Efforts to develop new GSSs for mosquitoes with phenotypes expressed as early as possible in the developmental process are continuous and diverse, employing both transgenic and non-transgenic approaches [14, 15, 23–26]. The quest for new marker genes has triggered the emergence of a new strategy called the generic (neo-classical) approach, which focuses on developing non-transgenic GSSs for SIT applications [24, 25]. Through the generic approach, genes and their specific mutations linked to desirable traits in a species are identified. The next step involves the induction of these mutations in orthologous genes of other species and the linkage of the wild-type allele to the male sex, which will result in a new GSS [26–30]. In the frame of this approach and because *ebony* is significantly closer to the sex locus than both *re* and *w* [31, 32], the *ebony* gene was selected as a candidate for gene editing in *Ae. aegypti* using CRISPR/Cas9 technology. *ebony* is involved in the pigmentation of *Drosophila* and encodes the enzyme N-β-alanyl dopamine (NBAD) synthetase, which converts dopamine to NBAD [33]. In *Ae. aegypti*, the disruption of the orthologous gene (AAEL005793) has been previously reported, and a prominent phenotype was observed at the larval and pupal stages [32]. In this study, we created a mutant *Ae. aegypti ebony* strain and evaluated its suitability and competence for the development of a new GSS for SIT applications.

## Methods

### *Aedes aegypti* strains and rearing conditions

The *Ae. aegypti* Liverpool (LVP) strain was used in all the experiments. The genome assembly AaegL5.0, which originated from the Liverpool strain, was also the reference genome used throughout the study [34]. Mosquitoes were reared in the insectaries of Virginia Tech and the Insect Pest Control Laboratory (Joint FAO/IAEA Centre, Seibersdorf, Austria) at 26–28 °C and 60–70% relative humidity with a 14/10 h day/night light cycle and 27 ± 1 °C, 80% relative humidity and a 12/12 h day/night photoperiod, respectively.

Larvae were fed a 4% liquid diet that included powdered yeast, tuna meal and black soldier fly [35], while adult mosquitoes were fed a 10% sucrose solution. At Virginia Tech, female mosquitoes were blood-fed defibrinated sheep’s blood (Colorado Serum Company, Denver, CO, USA) via artificial membrane feeders. At the IPCL, porcine blood was offered twice per week, and egg collection was initiated 72 h after the last blood feeding using moistened oviposition papers (white germination paper, Sartorius Stedium Biotech, Austria). The blood used was collected in Himberg, Austria, during routine slaughtering of pigs in a nationally authorized abattoir, conducted at the highest possible standards strictly following EU laws and regulations.

### CRISPR/Cas9-mediated knockout microinjections

The sgRNA 5’- GGCCGTGTTCGGCGCAACGC-3’ targeting exon 3 of the AAEL005793 gene was previously shown to successfully induce knockout of the *ebony* gene [32]. This sgRNA was synthesized by Synthego (www.synthego.com) with standard 2’ and 3’ base modifications and resuspended following the product guide (https://app.hubspot.com/documents/2418554/view/28153958?accessId=61e7dc). Approximately 500 embryos of the exu-Cas9 transgenic line [32] were injected with 100 ng/µl sgRNA using an Eppendorf FemtoJet injector. The surviving larvae and early pupae presented similar phenotypes in the head and siphon, as noted in [32]. Fourteen G_0_ adult survivors (six males and eight females) were mated together to increase the probability of generating homozygous individuals for easier screening of mutants in the following generation. The ebony phenotype was observed in 17 individuals at the larval and pupal stages of the ~ 500 total individuals screened. The line was maintained by crossing males and females exhibiting the ebony phenotype. The DsRed marker is the transformation marker for the exu_Cas9 line [32], which was used to generate the *ebony* knockout. We crossed DsRed-negative G_2_ individuals to generate a line without the exu-Cas9 transgene.

### Screening and genotyping

Larvae at L3–L4 stages were visually screened in every generation under a stereoscope, and non-ebony phenotypes (larvae that did not present dark heads and siphons) were removed. Non-lethal genotyping was initially performed at G_1_ (Virginia Tech) and then at G_7_ (IPCL) to determine the mutant genotype. For non-lethal genotyping, genomic DNA was extracted from single legs of adult mosquitoes following an adapted version of the protocol by [36]. Mosquitoes were anesthetized at 4 °C, and single legs were cut, placed in Eppendorf tubes and homogenized. The Platinum Direct PCR Universal Master Mix (Thermo Fisher Scientific) was used for DNA extraction and PCR amplification, following the manufacturer’s instructions. The primer pair used for confirming the *ebony* mutation was AAEL005793_01_F5’- TTTGGCCCTTGTTTAACCGA-3’ and AAEL005793_02_R 5’- AGAGACGTAAAGCCATTGAGATGT-3’. PCRs were performed in a 25-µl reaction volume and at the following PCR settings [94 °C, 2 min; 35 cycles of (94 °C, 15 s; 60 °C, 15 s; 68 °C, 20 s)]. The PCR amplicons were purified using the DNA Clean & Concentrator kit (Zymo Research) and Sanger sequenced (Eurofins Genomics, Germany). The sequencing trace files were processed with Geneious Prime 2022.0.2.

### Biological character assessment

The *Ae. aegypti ebony* mutant line at the fifth generation was compared with the wild-type LVP strain with respect to the following biological parameters:

Recovery rates: Eggs from both *Ae. aegypti* lines were hatched in airtight glass jars containing water with low dissolved oxygen content (boiled water). The jars were incubated in a climate chamber at 27 °C for 2 h. First-instar larvae (L1) were counted in batches of 100 and placed in trays containing 2 L of water and 30 ml of larval diet. For each line, we performed three replicates (*n* = 100 L1 per tray per line). Pupal and adult recoveries were recorded by counting the total number of pupae and adults, respectively.

Fecundity: For each strain, 20 newly emerged virgin females and 10 virgin males were placed in a BugDorm-4M2222 insect-rearing cage and mated for 3 to 4 days. Females were then blood-fed, and 3 days after the blood meal, plastic cups containing deionized water and lined with germination paper were provided in each cage for 48 h. The cages were monitored daily, and when dead females were noticed, they were replaced with other gravid females of the same age. Eggs were collected for the first two gonotrophic cycles, and the total number of eggs was counted under a stereoscope. Three replicates per strain were performed. Mean female fecundity was calculated by dividing the total number of eggs laid by all females by the total number of females.

### Statistical analysis

All statistical analyses were performed using R version 4.2.0 [37]. Pupal recovery and adult emergence rates represent recovery ratios; therefore, they were analyzed using a GLM-binomial family and a logit link function [38]. The fecundity data are count data and were analyzed with a generalized linear model (GLM) with Quasi-Poisson distribution and a log link function. The generalized linear model (GLM) overdispersion was checked with the DHARMA package [39]. Analysis of deviance was performed with a Chi-square test for the recovery and sex ratio rates and with an F test for the fecundity data [40]. In all boxplots, both the mean and the median values are depicted.

## Results

**Fig. 1:**
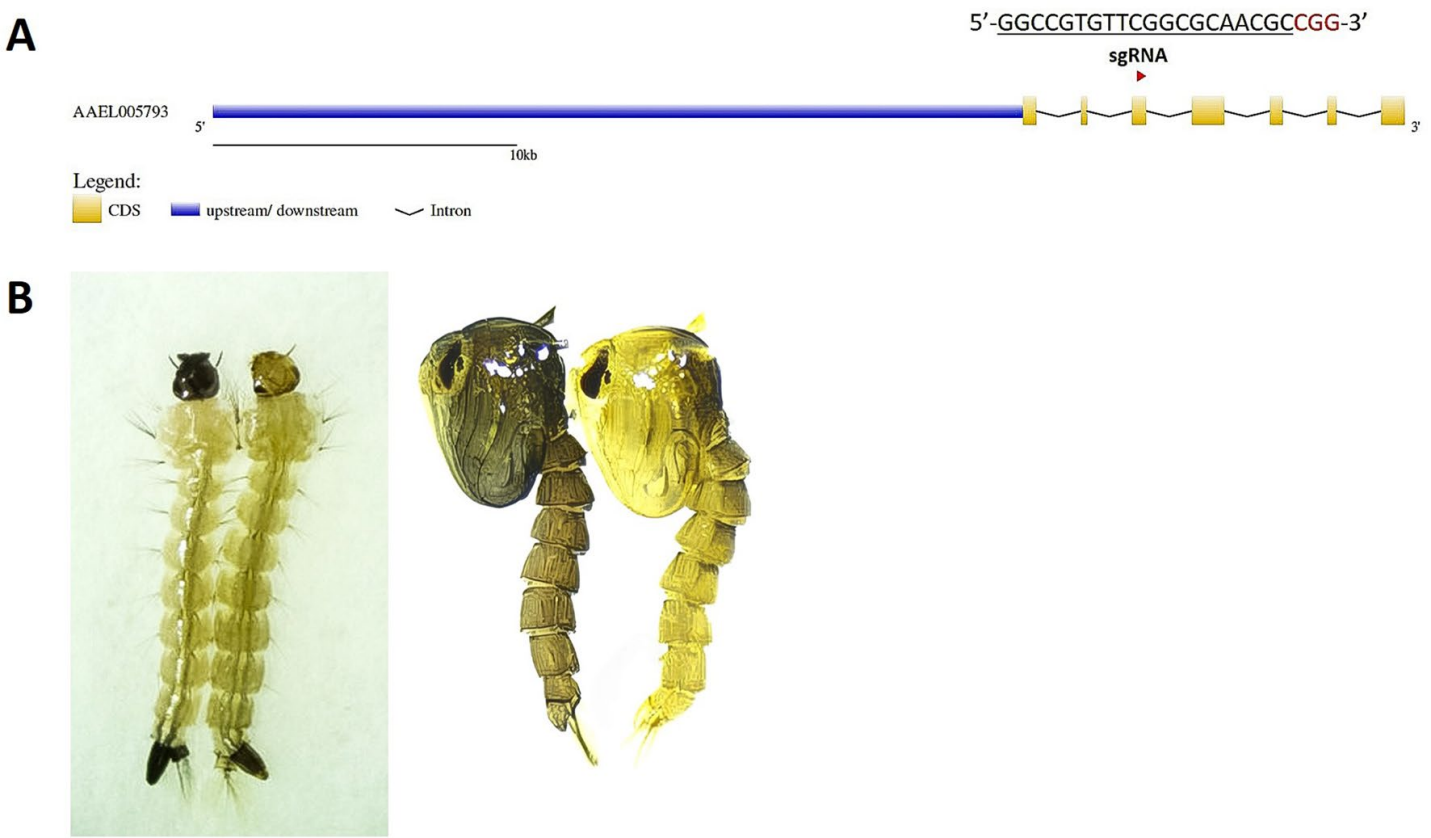
**A** Schematic representation of the *ebony* gene in *Aedes aegypti* spanning seven exons. The location and sequence of the sgRNA target [32] are indicated, with the PAM region shown in red. The scheme was designed using GSDS 2.0 [67]. **B** The ebony (left) and wild-type (right) phenotypes of larvae and 1-day pupae

The *Ae. aegypti ebony* gene is located in the recombination desert near the sex locus on chromosome 1 [31] and is 139,046 bp long. A total of seven exons constitute its coding region, which encodes a protein of 884 amino acids (Fig. 1a). A sgRNA targeting the third exon of the *Ae. aegypti ebony* gene was injected into approximately 500 LVP embryos. G_0_ individuals who survived the injection process were screened at the larval stage. Fourteen G_0_ males and females exhibiting the ebony phenotype presented dark larval heads and darker heads at the pupal stage (Fig. 1b). These 14 individuals were inbred to form a homozygous *ebony* line without being previously genotyped. Our results confirm the phenotypes previously described in *Ae. aegypti* by [32].

**Fig. 2:**
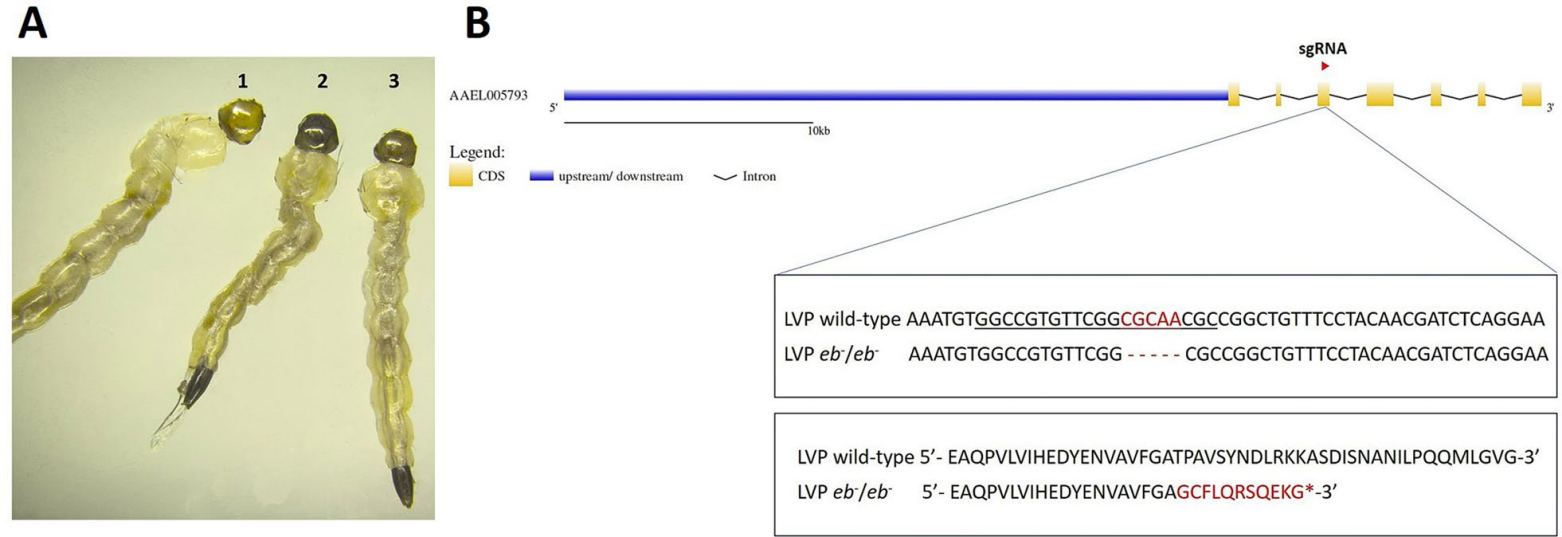
**A** Wild-type (1), ebony (2) and intermediate (3) phenotypes in larvae. **B** Sanger sequencing showed a 5-bp deletion (in red) in homozygous adults. The sgRNA sequence is underlined. The deletion results in an early stop codon and yields a shorter protein of only 161 amino acids (in red) compared to the 884 amino acids of the wild-type protein

During larval screening at G_6_, mixed phenotypes, which included the ebony (black larval heads), intermediate (dark brown larval heads) and wild-type phenotypes (Fig. 2a), were detected. The intermediate ebony phenotype exhibited phenotypic inconsistency by showing a spectrum of pigmentation levels. Thirty-six larvae with the ebony phenotype were set apart to re-establish a pure *ebony* colony. G_7_ individuals were screened both phenotypically and genotypically. A 5-bp deletion in exon 3 was detected, resulting in an early stop codon and yielding a shorter protein of only 161 amino acids (Fig. 2b and Additional file 1: Fig. S1). Phenotype screening, single-leg PCR, Sanger sequencing and crosses were performed during the next generations (starting from G_8_) to isolate mosquitoes carrying the *eb*^−^/*eb*^−^ genotype and create a homozygous line. The number of genotyped males and females per generation is shown in Additional file 2: Table S1.

**Fig. 3:**
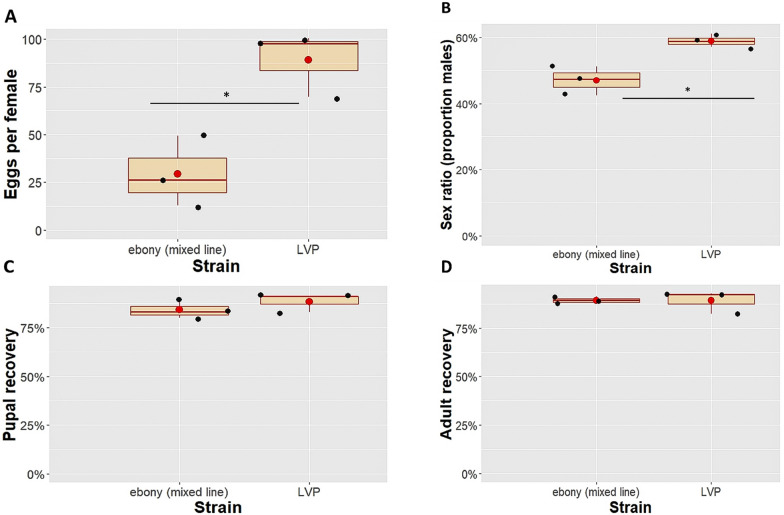
**A** Effect of *ebony* mutation on female fecundity. The number of eggs per female is shown on the y-axis for the *ebony* and LVP groups. The fecundity of the two lines differs significantly (*P* = 0.0232). **B** Sex ratio of males produced from the *ebony* and the LVP groups. The *ebony* line had a significantly lower male proportion (*P* = 0.01115). **C, D** Effect of *ebony* mutation on pupal and adult recovery. No significant difference in pupal (*P* = 0.1236) or adult (*P* = 0.9332) recovery was detected. In all the cases, the boxplots span the interquartile range, and the whiskers indicate the highest and lowest observations. The red line and the red dot inside each box represent the median and the mean, respectively. The black dots represent the three different replicates. Significant differences between treatment groups are indicated with asterisks (***p < 0.001, **p < 0.01, *p 0.05, ns: *p* > 0.05; confidence level used: 0.95, alpha = 0.05)

As shown in Table 2, genotyping at G_7_ revealed that the wild-type phenotype can occur in all possible genotypes (+/+, + /*eb*^−^ and *eb*^−^/*eb*^−^). Larvae expressing the intermediate phenotype could be either heterozygotes or homozygotes for the deletion, while larvae with the ebony phenotype were always homozygotes for the deletion. During the next generations (from G_8_ to G_13_), female and male homozygotes for the deletion were selected and crossed to create an *eb*^−^ homozygous line. All the selected males expressed the ebony phenotype, while all the selected females expressed the intermediate phenotype. Crossing of the homozygous adults led to zero progeny or to a minimum number of eggs that did not hatch. The homozygous line could not be maintained, and it was eventually lost. This restriction in maintaining a clear homozygous mutant line was further confirmed by the quality control results. Quality tests were performed at G_5_ before we verified that our line was not genotypically pure. Despite the presence of all three genotypes (although the ratio among them is not known), we observed a fitness cost of the mixed line compared with the wild-type LVP strain regarding fecundity (ANOVA, *F*_*(1, 4)*_ = 12.806, *df* = 1, *P* = 0.0232) and the sex ratio (Chi-square test,* χ*^*2*^ = 1.4347, *df* = 1, *P* = 0.01115) (Fig. 3A, B). The pupal and adult recovery rates did not differ significantly (pupae: ANOVA, *F*_*(1, 4)*_ = 7.1871, *df* = 1, *P* = 0.1236; adults: ANOVA, *F*_*(1, 4)*_ = 6.5424, *df* = 1, *P* = 0.9332) (Fig. 3C, D). The cost conferred by the *ebony* mutation, either in homozygosity or heterozygosity, was later validated by our unsuccessful efforts to create a pure homozygous mutant line.

## Discussion

Insect body coloration is formed mainly by melanin, a pigment group that is synthesized, metabolized and transported by a complex set of genes [41]. *ebony* has been described as one of these genes involved in epidermal pigmentation and promoting light-color cuticle tanning [42–47]. Body pigmentation can determine how insects interact with the environment and guide their adaptation and evolution [42, 47, 48]. *ebony* has been widely studied in insects not only because of its profound phenotype when mutated but also because of its conserved function across phylogenetically distant insect species, including *Diptera* and *Lepidoptera* [49]. At the protein level, Ebony is highly conserved among species (Table 1), which underscores its essential role in body coloration.

**Table 1:** Protein alignment (% identity) of Ebony in several insect species

	*Aedes* *aegypti* (%)	*Aedes* *albopictus* (%)	*Anopheles* *gambiae* (%)	*Drosophila melanogaster* (%)	*Bombyx mori* (%)
*Aedes aegypti*		94.6	73.1	57.2	47
*Aedes albopictus*	94.6		73.3	57.4	47.2
*Anopheles gambiae*	73.1	73.3		58.9	48.2
*Drosophila melanogaster*	57.2	57.4	58.9		47.5
*Bombyx mori*	47	47.2	48.2	47.5	

*ebony* is considered a melanin-inhibiting gene and takes part in the melanism pathway, along with *yellow*, which promotes melanin synthesis [33, 47]. Both genes display visible pigmentation changes when mutated, with *yellow* mutants having a lighter body color and *ebony* a darker body phenotype [32, 47]. Mutations in *yellow* genes in insects have also been associated with fitness costs that include reduced male mating success due to altered copulatory structures and impaired locomotor performance [50, 51]. These fitness costs highlight the critical role of these genes not only in cuticle pigmentation but also in the strain’s viability and performance.

Similar to our results in *Ae. aegypti*, *ebony* mutants in other insect species showed a darker body phenotype that, depending on the case, was to a certain extent evident at the larval, pupal or adult stage [32, 42, 47, 49, 52, 53]. In *Drosophila melanogaster* and *Plutella xylostella*, heterozygous *ebony* mutants exhibited incomplete melanization, while complete melanization was evident only in individuals homozygous for the deficiency [49, 54]. The intermediate phenotype exhibited in these two species indicates a semi-dominant character of *ebony*, which, however, was not confirmed in a *Bombyx mori* study [42] or in *Ae. aegypti* (this study). In *B. mori*, all heterozygotes for the mutation presented the wild-type phenotype, and homozygous mutants were completely melanized. On the other hand, in *Ae. aegypti*, an intermediate phenotype was also present at the larval and pupal stages in both males and females, but the genotypes were either homozygous (*eb*^−^/*eb*^−^) or heterozygous (+ /*eb*^−^) for the mutation. *Aedes aegypti* exhibiting the wild-type phenotype added extra layers of complexity since they had all possible genotypes (+/+, +/*eb*^−^, *eb*^−^/*eb*^−^). Unlike the previously studied *Diptera* and *Lepidoptera*, it seems that, at least in *Ae. aegypti*, the *ebony* mutation is not fully penetrant and expressive. Incomplete penetrance and variable expressivity are an interplay of various factors, including but not limited to interactions of *ebony* with other genes, the genetic background of the strain used to develop the mutation, the presence of regulatory factors that influence the expression of the gene as well as the position of the gene itself and the chromatin structure of the area [55]. However, it cannot be excluded that a different *ebony* mutation in the promoter region or in another exon might present a different expression and penetrance profile that could lead to a viable homozygous mutant line.

Despite the high conservation of the Ebony protein across insect species, the effects caused by a gene mutation can vary among different genomic and environmental backgrounds [56, 57]. The ebony phenotype has been extensively described in several mosquito species, which exhibit a variety of expression patterns. In *Anopheles albimanus*, the homozygous larvae are uniformly black, but they die before they transform into pupae. Conversely, heterozygotes present an intermediate phenotype but are fully viable [45]. The same black larval phenotype has also been described in *Culex tritaeniorhynchus* [58], while in *Culex quinquefasciatus*, the dark phenotype is expressed only at the larval head [59], similar to *Ae. aegypti*. In *Culex tarsalis* adult *ebony* mutants, white scale bands from the proboscis, legs and abdomen are lost [44]. This lack of universality in mutant ebony phenotypes could be attributed to epistasis phenomena that can lead to different phenotypic outcomes due to complex gene interactions among species [60]. The sister species *Drosophila yakuba* and *Drosophila santomea* differ in terms of abdominal pigmentation due to cis-regulatory changes. In *D. yakuba, ebony* expression is restricted to the anterior abdominal segments, while in *D. santomea,* it is expressed along the anterior-posterior axis [61]. Cis-regulatory mutations can alter the gene network topology as well as any direct or indirect gene interactions. On the other hand, mosquito mutants discovered and characterized decades ago might not result from mutations of “*ebony*” gene. Thus, the mutants characterized as “*ebony*” must be treated cautiously to avoid any misleading conclusions.

Pleiotropy is a common trait among genes that determine body coloration. *ebony* is involved in a wide range of biological functions, including cuticular hydrocarbon composition, mating behavior, vision, circadian rhythm and innate immunity, in *Drosophila* species [43, 51, 62–64]. Knockout mutants of *ebony* can therefore affect multiple discrete phenotypic traits and confer fitness costs related to either mild or detrimental effects. In *D. melanogaster*, homozygous mutant male flies are partially blind and suffer from low mating competitiveness [65]. In *Cx. quinquefasciatus*, CRISPR attempts to create *ebony*-knockout mutants led to either G_0_ lethality or a low number of survivors [59]. In *A. albimanus*, complete lethality has been recorded for homozygous mutants before they reach the pupal stage, while lower hatchability and larval survival rates have been reported in *P. xylostella* [45, 49]. In *Ae. aegypti*, female pupae expressing the ebony phenotype were scarce in every generation (no more than one or two individuals per generation), and they were all genotyped. All of them were homozygotes for the deletion and died at the pupal stage. On the other hand, the homozygous females expressing the intermediate phenotype survived to adulthood and were used for the crosses. Interestingly, all the homozygous male mosquitoes expressing the ebony phenotype survived to adulthood (Table 2). In addition, crosses among homozygous adults led to non-viable embryos or the complete absence of egg laying. Our data indicate that complete melanization, and not the homozygosity, is lethal to female but not to male *Ae. aegypti*, which can be linked to the pleiotropic nature of the gene [64]. Excessive production of pigments might interact with sex-linked genes and lead to early female lethality; however, this hypothesis requires further investigation.

**Table 2:** Genotyping of G_7_ individuals expressing the wild-type, intermediate and ebony phenotypes

Code	Sex	Phenotype	Genotype
1	F	Wild type	± + / -
2	F	Wild type	± +/ -
3	F	Wild type	± + / -
4	F	Wild type	± + / -
5	F	Wild type	+ / +
6	F	Wild type	± + / -
7	F	Wild type	+ / +
8	F	Wild type	+ / +
9	F	Wild type	+ / +
10	M	Wild type	−/−
11	M	Wild type	−/−
12	M	Wild type	−/−
13	M	Intermediate	−/−
14	M	Intermediate	−/−
15	M	Intermediate	± + / -
16	M	Intermediate	± + / -
17	M	Intermediate	−/−
18	M	Ebony	−/−
19	M	Ebony	−/−
20	M	Ebony	−/−

Identifying genes responsible for phenotypic variation in insects can reveal promising targets that could be used for pest management in SIT applications. These targets can be disrupted through genome editing techniques, such as CRISPR/Cas9 technology, and can be used for the construction of a new GSS. Marker genes should fulfill certain criteria to be considered eligible for GSS development. They should be easily identifiable and should not impose fitness costs on the species. *ebony* is a well-studied gene that has been edited via CRISPR in various species, including *Ae. aegypti* [32]. In *Spodoptera litura*, *ebony* has been suggested as a promising candidate for genome editing and control strategies [53]. On the other hand, it has been recently shown that disruption of *ebony* in *Bactrocera tryoni* results in reduced hatchability and eclosion [66]. In this study, we showed that the *ebony* mutant we created is not suitable for the development of an *Ae. aegypti* GSS because of its phenotypic inconsistency and the loss of fertility of the homozygous mutant line.

## Conclusions

*Aedes aegypti* is widely distributed in tropical and subtropical regions, and it is able to breed in human-made breeding sites. *Aedes aegypti* is also expected to invade and establish in previously unoccupied areas due to the high volatility of climate conditions. The development of a GSS that minimizes the possibility of accidental female release is critical to the environmentally friendly and sustainable SIT approaches for the control of *Ae. aegypti* populations. In this study, we evaluated whether the ebony phenotype could serve as a selectable marker for the development of a GSS. The results revealed that the *ebony* mutation we developed results in reduced penetrance and expressivity and presents fertility loss in the homozygous state. Therefore, it cannot be recommended as a robust and reliable selectable marker, and additional mutants or genes need to be investigated to identify suitable markers.

## Supplementary Information

Supplementary Materials 1. Fig. S1. *ebony* gene Sanger sequencing of wild-type, homozygous and heterozygous individuals.

Supplementary Materials 2. Table S1: Number of genotyped males and females per generation.

## Data Availability

All data of this study are available in the manuscript, its associated supplementary files, and upon request.

## Abbreviations

GLM: Generalized linear model
GSS: Genetic sexing strain
IPCL: Insect pest control laboratory
LVP: Liverpool
NBAD: N-β-alanyl dopamine
SIT: Sterile insect technique
