# Supplementary figures and images for: Evaluation of ebony as a potential selectable marker for genetic sexing in Aedes aegypti
Source: Parasit Vectors. 2025 Feb 25;18:76. doi: 10.1186/s13071-025-06709-y (PMC11863432; doi:10.1186/s13071-025-06709-y)

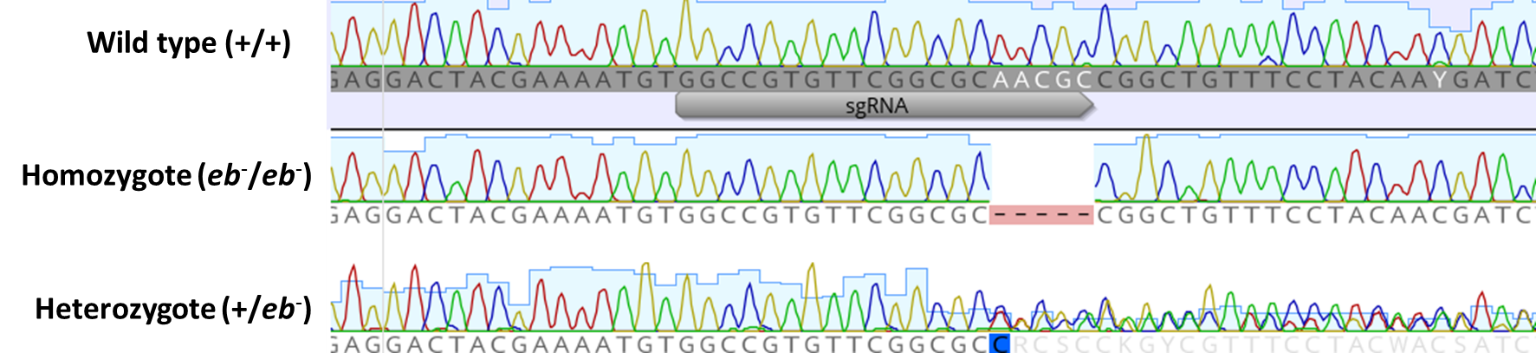


**Figure S1**: *ebony* gene Sanger sequenced in wild type, homozygote and heterozygote individuals.

Supplement: Supplementary file 1 — Supplementary Materials 1. Fig. S1. ebony gene Sanger sequencing of wild-type, homozygous and heterozygous individuals. [file 13071_2025_6709_MOESM1_ESM.docx]
