# Supplementary material for: Evaluation of ebony as a potential selectable marker for genetic sexing in Aedes aegypti
Source: Parasit Vectors. 2025 Feb 25;18:76. doi: 10.1186/s13071-025-06709-y (PMC11863432; doi:10.1186/s13071-025-06709-y)
Supplement: Supplementary file 2 — Supplementary Materials 2. Table S1: Number of genotyped males and females per generation. [file 13071_2025_6709_MOESM2_ESM.docx]

**Table S1**: Number of genotyped males and females per generation.

| **Generation** | **G_7_** | **G_8_** | **G_9_** | **G_10_** | **G_11_** | **G_12_** | **G_13_** | **Total** |
| --- | --- | --- | --- | --- | --- | --- | --- | --- |
| **Females** | 9 | 0 | 6 | 7 | 6 | 4 | 8 | **40** |
| **Males** | 11 | 16 | 4 | 0 | 6 | 13 | 3 | **53** |
